# Susceptibility to a sexually transmitted disease in a wild koala population shows heritable genetic variance but no inbreeding depression

**DOI:** 10.1111/mec.16676

**Published:** 2022-09-12

**Authors:** Romane H. Cristescu, Kasha Strickland, Anthony J. Schultz, Loeske E. B. Kruuk, Deidre de Villiers, Céline H. Frère

**Affiliations:** ^1^ Global Change Ecology Research Group University of the Sunshine Coast Sippy Downs Queensland Australia; ^2^ Institute of Ecology and Evolution University of Edinburgh Edinburgh UK; ^3^ Icelandic Museum of Natural History (Náttúruminjasafn Íslands) Reykjavik Iceland; ^4^ Research School of Biology Australian National University Canberra Australian Capital Territory Australia; ^5^ Endeavour Veterinary Ecology Toorbul Queensland Australia; ^6^ School of Biological Sciences University of Queensland St Lucia Queensland Australia

**Keywords:** additive genetic effect, *Chlamydia pecorum*, inbreeding, *Phascolarctos cinereus*, quantitative genetics

## Abstract

The koala, one of the most iconic Australian wildlife species, is facing several concomitant threats that are driving population declines. Some threats are well known and have clear methods of prevention (e.g., habitat loss can be reduced with stronger land‐clearing control), whereas others are less easily addressed. One of the major current threats to koalas is chlamydial disease, which can have major impacts on individual survival and reproduction rates and can translate into population declines. Effective management strategies for the disease in the wild are currently lacking, and, to date, we know little about the determinants of individual susceptibility to disease. Here, we investigated the genetic basis of variation in susceptibility to chlamydia using one of the most intensively studied wild koala populations. We combined data from veterinary examinations, chlamydia testing, genetic sampling and movement monitoring. Out of our sample of 342 wild koalas, 60 were found to have chlamydia. Using genotype information on 5007 SNPs to investigate the role of genetic variation in determining disease status, we found no evidence of inbreeding depression, but a heritability of 0.11 (95% CI: 0.06–0.23) for the probability that koalas had chlamydia. Heritability of susceptibility to chlamydia could be relevant for future disease management, as it suggests adaptive potential for the population.

## 1 INTRODUCTION

Disease can pose a serious threat to flora and fauna, recently demonstrated through the global impact of white nose syndrome (Hoyt et al., 2021), sarcoptic mange (Escobar et al., 2021), and anthrax (Hoffmann et al., 2017). Similarly, plant diseases such as chestnut blight and Dutch elm disease have had large‐scale impacts on species and ecosystem diversity (Burke, 2012; Strobel & Lanier, 1981). It is also likely that threats to wildlife populations from disease will increase in frequency, due to interactions with both climate change and accelerating anthropogenic disturbances (Daszak et al., 2000; Harvell et al., 2002; Hassell et al., 2017). In addition to the threat to biodiversity, wildlife diseases can have substantial economic impacts and affect ecosystem services (Sleeman, 2013). Wildlife diseases also pose a growing threat to human health, with an estimated 75% of emerging infectious diseases in humans being of wildlife origin (Merianos, 2007).

Managing diseases in wild populations is difficult, in part because effective management of disease requires an understanding of host‐pathogen‐environment dynamics that may be costly to obtain (vander Wal et al., 2014). Intensive management such as vaccination might only be feasible in certain situations, where vaccines can be distributed in baits that are readily taken (e.g., rabies vaccine for carnivores: Slate et al., 2009) or for small, contained populations (Woodroffe, 1999). Targeted disease mitigation strategies in the case of the chytrid fungus in amphibians, for instance, have included preventing pathogen spread to naive populations, establishing ex situ insurance colonies, and captive breeding of amphibian hosts (Fisher & Garner, 2020). To achieve effective management of already‐infected populations, however, mitigation strategies need to integrate specific host‐pathogen interactions, in particular targeting pathogenicity and host susceptibility (Woodhams et al., 2011). Moreover, as part of the increased appreciation of the value of evolutionary insights for wildlife disease management, there is increasing awareness of the need to understand what factors contribute to susceptibility to disease. This information can be fundamental in predicting whether species and populations will adapt to diseases, and could enable us to assist this process by selecting resilient animals (Blanchong et al., 2016; McKnight et al., 2017).

Many factors influence the susceptibility of a host to a pathogen. The host's environment will have multiple effects on its susceptibility to disease: for example, the quality and quantity of food resources can influence host condition and hence immunological health (Martin II et al., 2008; Smith, 2007), and both the prevalence of the pathogen and characteristics of individuals' social environment can affect infection risk (Caillaud et al., 2006). Host genetics may also play a role in determining individuals' suceptibility to disease (Parker et al., 2014; Trang et al., 2019). For example, genetic variation may affect susceptibility via additive genetic effects (Hill, 2012). Another genetic characteristic related to the ability of a host to cope with disease is inbreeding, the risk of which increases with small populations. Links between inbreeding and lowered disease resilience (i.e., the cumulative impact of resistance and tolerance) may be driven by inbreeding depression, explained through decreased heterozygosity in genes of the immune system (e.g., major histocompatibility complex (O'Brien & Evermann, 1988)), and the loss of alleles linked to resilience (Spielman et al., 2004). As such, determining the extent of heritability of susceptibility to disease and potential inbreeding depression linked to the disease could be valuable for disease management. In particular, a genetically heritable basis to variation in susceptibility indicates the potential for evolutionary responses, and for selective breeding for resistance, whereas the existence of strong effects of inbreeding would support efforts to improve genetic variance within populations.

In this study, we present quantitative genetic and inbreeding depression analyses of a disease affecting an Australian mammal, the koala *Phascolarctos cinereus*. Recent declines in population size, including those due to extreme bushfires in 2020, have led to the classification of koalas in Queensland, New South Wales, and the Australian Capital Territory (approximately 60% of their range) as endangered in 2022. Whilst the bushfires decimated large areas of koala habitat, the bacterial pathogen *Chlamydia* has been identified as a major threat to their survival (McAlpine et al., 2015). The bacterium causes chlamydial dNcKnight disease, which primarily affects the ocular and urogenital tracts in koalas and decreases both survival and reproduction rates (Brown et al., 1987). The main transmission path is sexual, although some mother‐offspring transmission does occur (Nyari et al., 2017). The severity of chlamydial disease varies greatly both between and within populations (Ellis et al., 1993; Waugh, Hanger, et al., 2016), and not all koalas that are infected progress to “diseased” status (i.e., show clinical signs of disease) (Robbins et al., 2019). Chlamydial disease is the most common illness causing death of wild koalas (Gonzalez‐Astudillo et al., 2017) and is such a critical threat to koalas that, when adequately addressed, population declines can be reversed (Beyer et al., 2018; Rhodes et al., 2011). However, management of the disease currently requires costly treatment, including catching koalas and multiple weeks of antibiotic treatment at wildlife hospitals (Beyer et al., 2018). An understanding of the potential for evolutionary responses could therefore greatly improve management strategies for koala populations.

Using high‐resolution data from a wild population of koalas, we investigated the effect of genetic and environmental factors on chlamydia disease. There is a high prevalence of chlamydia disease in the study population (28%: Hanger et al., 2017), and chlamydia is a leading cause of mortality (18% of all deaths: Beyer et al., 2018). Further, we have previously demonstrated that koalas in this population do not avoid mating with either diseased, or with closely‐related individuals (Schultz et al., 2020). Here, we aimed to (1) determine whether there was evidence for inbreeding depression associated with disease susceptibility, and (2) partition variance in susceptibility to disease into additive genetic and environmental components. Our analysis provides insight into the extent to which an individual's genes and/or environment affects their susceptibility to disease, and whether a wild population could respond to selection for improved resilience to chlamydial disease.

## 2 MATERIALS AND METHODS

### 2.1 Study population

Our study population consisted of 519 koalas that were monitored between 2013 and 2017 as part of a management programme associated with a 13 km rail infrastructure project in Moreton Bay Council, Queensland, Australia (area centroid: −27.234°; 153.036°), and described in detail in (Hanger et al., 2017). During the 5‐year programme, most of the population (>95% of individuals in the study area) was intensively monitored. Individuals were first captured between 13/03/2013 and 02/02/2017 using either live traps or flagging pole methods, and were fitted with VHF collars. After their first capture, individuals were then periodically recaught at least every 6 months for routine veterinary exams, or earlier if they were observed with visible signs of disease (see below) or injury. Tissue samples were taken and ear tags fitted at first capture, and blood samples were taken during routine veterinary examinations, at first capture and at subsequent check‐ups. Blood samples were stored at −20°C, and tissue samples were stored in 70% ethanol. Individuals were given a comprehensive veterinary examination each time they were caught, which included recording of sex, age, and presence of a joey (offspring) for females (either through the female having a back‐riding or pouch joey or an elongated teat, or through a pregnancy observed by sonogram). Aging of koalas relied on examination of their tooth wear (Gordon, 1991). They were then tested for the presence of chlamydia bacterium and inspected for visible signs of chlamydial disease (see below for full details). During the project, individuals were treated for any illness (including chlamydial disease, see below) or injury, and some individuals were also included in a chlamydia vaccination trial, which involved a subset of individuals being vaccinated for chlamydia at their first capture (see Waugh, Khan, et al., 2016). VHF tracking of individuals took place approximately twice a week. By identifying the time of the year in which joeys were born, and accounting for the gestation period (1 month), based on the birth of 350 joeys between 2013 and 2016, we determined the breeding season to occur from September to December in this population. The analyses presented here used data from those individuals for which we had complete genetic, spatial and disease status data (described below, *N* = 342). The data set included some joeys (*N* = 4) because dependent joeys have been found to be infected by chlamydia as young as 9 months old (Nyari et al., 2017) and the youngest dependent joey sampled in this survey was 10 months old.

The monitoring programme was conducted under animal ethics approvals (Queensland Department of Agriculture and Fisheries CA 2012/03/597, CA 2013/09/719, CA 2014/06/777, CA 2015/03/852, and CA 2016/03/950) and scientific purposes permits (Queensland Department of Environment and Heritage Protection WISP 11525212, WISP 16125415, WISP 13661313, WITK 14173714 and WISP 17273716).

### 2.2 Chlamydial disease

Of the two species of chlamydia that infect koalas (*Chlamydia pecorum* and *C. pneumoniae*), *C. pecorum* is consistently more prevalent (i.e., higher percentage individuals infected) and more pathogenic (Jackson et al., 1999; Polkinghorne et al., 2013). *C. pecorum* is therefore the chlamydia species that researchers and veterinarians focus on (Quigley et al., 2018). At each veterinary examination, koalas were (1) tested for the presence of *C. pecorum*, and (2) assessed for clinical signs of chlamydial disease. Tests for *C. pecorum* were conducted using either quantitative real‐time PCR (qPCR) targeting a fragment of the *C. pecorum* 16 s rRNA gene (Marsh et al., 2011), or using the Clearview Chlamydia MF test kit, which has a 60% sensitivity (Inverness Medical, Unipath Ltd). Clinical signs of chlamydial disease are mostly found in the ocular and urogenital regions. The ocular form of the disease varies from inflammation of the mucosal surfaces of the eye, or conjunctival hyperplasia, to the complete opacification and scarring of the cornea, which can cause blindness (Cockram & Jackson, 1981). In urinary disease, inflammation of the bladder (cystitis) leads to incontinence and staining of the fur around the cloaca, which is also coupled with alopecia and ulceration in severe cases (Burach et al., 2014). Finally, infection of the reproductive tract in females causes bursal cysts surrounding the ovary and upper reproductive tract pathology, while in males orchitis and epididymitis can occur (Johnston et al., 2015; Polkinghorne et al., 2013). In this study, veterinary diagnostic techniques for assessing the presence of chlamydial disease involved observation of external signs of disease, cystocentesis with observation of the urine sediment, and ultrasound examination of the kidneys, reproductive tract, and bladder (as described in (Robbins et al., 2019)). We initially aimed to independently analyse whether koalas tested positive for the presence of *C. pecorum*, and whether koalas were diagnosed with clinical signs of disease. However, in our data, the majority of individuals that tested positive also displayed clinical signs of disease (87%). We therefore opted to use whether koalas tested positive (“chlamydia status”) as our response variable in all analyses.

Although individuals were monitored for multiple years (average = 1.2 years, max = 4 years) resulting in repeated measures of disease status gathered at scheduled veterinary examinations (*N* = 1182 observations of *N* = 342 koalas, average = 3 observations per koala), we selected to only use chlamydia status at their first capture. This was because during the project individual koalas were treated for any illness or injury they presented at veterinary examinations (including chlamydial disease), and some individuals were also included in a chlamydia vaccination trial (Waugh, Khan, et al., 2016). Individual disease status at subsequent veterinary examinations could therefore have been affected by the veterinary treatment, vaccine trial and management of the population.

### 2.3 DNA extraction and genotyping

We conducted DNA extraction and genotyping as previously described for this population (Schultz et al., 2020). Briefly, we used single nucleotide polymorphism (SNP) genotyping data from blood or tissue samples. DNA was extracted using the DNeasy Blood and Tissue Kit (Qiagen), following the manufacturer's protocol, and DNA extracts were stored at −80°C. Genotyping was conducted as per (Kjeldsen et al., 2018) by Diversity Arrays Technology, Canberra, using their proprietary DArTseq technology. DArTseq utilizes a combination of next‐generation sequencing platforms and DArT complexity‐reduction methods (Cruz et al., 2013; Kilian et al., 2012), and, similar to DArT methods based on array hybridizations, the protocol is optimized for a specific organism and application by selecting the most appropriate complexity reduction method. This is assessed based on minimizing skewed size ranges, nonideal numbers of fragments, and percentages of repetitive elements. Samples were then processed as per Kilian et al. (2012). Genotyping produced a total of 8649 SNPs, which were then filtered to remove SNPs with MAF < 1%; call rate <95%; technical replicate scores <95%; and HWE where *p* > .05. This resulted in a total of 5007 SNPs that were used for subsequent analyses. Observed and expected heterozygosity, as well as the inbreeding coefficient *F*_IS_, were calculated using the dartR package (Gruber & Georges, 2018) following the same procedure as in Schultz et al. (2022).

### 2.4 Relatedness assignment

We calculated pairwise relatedness estimates using the R package Related (Pew et al., 2015) using the filtered set of 5007 SNPs, as per Schultz et al. (2020). Pairwise relatedness across all pairs of individuals in our genetic data set was estimated using Queller and Goodnight's (1989) relatedness estimator. Although there are multiple relatedness estimates available, we selected to use that of Queller and Goodnight because it has previously been found to be the most accurate for this data set in determining true pairwise relationships. Briefly, using simulated genotypes of known relationships, Schultz et al. (2020) compared the accuracy of different estimates in correctly determining relationships (i.e., parent‐offspring or half‐sibs), finding that the estimate of Queller and Goodnight was the most accurate. Maternities were identified through genetic parentage analyses, or, rarely, through field observations. Materinity assignment was conducted using a reduced panel of more stringently filtered SNPs (*N* = 427 SNPs, MAF > 0.3, missingness <1%, consistent marker scores for technical replicate assay pairs of >99%), linkage disequilibrium (*r* ^2^ < .4) in Cervus 3.0.7 as per Schultz et al. (2020).

### 2.5 Inbreeding coefficients

Using the same SNP set (*n* = 5007 SNPs) as for pairwise relatedness, we calculated “internal relatedness” (*IR*) for each individual (Amos et al., 2001). This measure was chosen as it strongly correlates with standard measures of heterozygosity but incorporates further information about the frequency of the alleles in the individual's genotype. As such, internal relatedness is a common measure of individual heterozygosity used to investigate inbreeding depression. When calculated over multiple loci, individuals' internal relatedness values in a population are approximately normally distributed with negative values suggesting more “outbred” individuals and positive values being suggestive of inbreeding. We used the Genhet version 3.1 function (Coulon, 2010) in the R statistical environment to estimate IR as $\mathrm{IR}=\left(2H-\sum \mathrm{fi}\right)/\left(2N-\sum \mathrm{fi}\right)$, where *H* is the number of loci that are homozygous, *N* is the number of loci and fi is the frequency of the *i*th allele contained in the genotype.

### 2.6 Shared environment effects

Phenotypic similarity between individuals in any quantitative trait may be generated by individuals experiencing similar environmental conditions, as well as by their shared genes (Kruuk & Hadfield, 2007). This may include, for example, effects of variation in resources (e.g., habitat quality) or of social networks (two individuals with spatial overlap will be more likely to encounter the same conspecifics). We accounted for the possible effects of shared environments by estimating individuals' overlap in their spatial environments, using data on their home ranges (see model details below). Home ranges were estimated as utilization distributions using VHF radiotracking locations of collared koalas. Tracking data was therefore collected after our measure of infection (i.e., whether koalas tested positive at first capture). However, koala home ranges are thought to be relatively stable through time, even when disturbed by habitat changes (Kavanagh et al., 2007; Matthews et al., 2016). Utilization distributions describe the frequency distribution of individuals' location data, and estimate the probability of an individual occurring across the study site (Fieberg & Kochanny, 2005). We included all individuals with at least five sightings over the study period (*N* = 342). The average number of sightings per individual was 186 (interquartile range = 48–346). Utilization distributions were estimated using the adehabitatHR package (Calenge, 2006) in the R statistical environment (R Core Team, 2020) with a smoothing parameter of 100 m and grid size of 50 m. This smoothing parameter was selected to (1) standardize the estimation of home ranges of all individuals, and (2) avoid over‐ or under‐smoothing in utilization distribution estimation for individuals with few sightings. All utilization distributions were visually inspected post estimation in order to ensure accuracy. Home range overlap (abbreviated “HRO” in the results) between all pairs of individuals was then measured using utilization distribution overlap indices (Fieberg & Kochanny, 2005), from which a home‐range overlap matrix was constructed. This measures the extent to which koalas overlap spatially, although it does not account for the possibility that some pairs of koalas may co‐occur spatially but not temporally.

### 2.7 Statistical analysis

We first tested whether our genetic data set contained the power to detect inbreeding (if present) using the R package inbreedR to estimate identity disequilibrium (*g*_2_: Stoffel et al., 2016). Specifically, this tested for variance in inbreeding in the population, which is required to detect inbreeding depression. Then, to investigate whether there was evidence for inbreeding depression, additive genetic effects, and/or shared environment effects, we ran a suite of generalized linear mixed effects models using the MCMCglmm package (Hadfield, 2012) in the R statistical environment. In all models, our response was a binary variable describing whether or not the koala tested positive for *C. pecorum* at first capture (effectively a case/control comparison), and all models were fit with the following fixed effects: age in years at time of capture, season in which the individual was caught (breeding vs. nonbreeding), sex, IR, and the interaction between age and IR. The effect of IR tested for evidence for inbreeding depression, predicting that if there was evidence for inbreeding depression, more inbred individuals would have poorer health outcomes and would therefore be more likely to test positive for *C. pecorum*. We also included an interaction between IR and age to test whether the effect of inbreeding changes with age (Marjamäki et al., 2021). Although it is likely that the components of variance also change with age, our data set structure and size were not suitable for addressing this question: of the 342 individuals in the data set, only 20 individuals were between 0–1 years old, and 81 between 1–2 years old, resulting in very few observations of individuals prior to sexual maturity, making estimating variances at different ages difficult using this data set. Fixed effects were given flat weakly informative priors, and random effects were given a $\chi_{1}^{2}$ prior, following the advice of (de Villemereuil et al., 2013). In this study, de Villemereuil and colleagues found that when estimating heritability of binary traits, this prior was less sensitive to the inclusion of multiple random effects than alternative priors and performs best with small sample sizes. We ran 1,030,000 iterations per model with a burnin period of 30,000 iterations, sampling at intervals of 1000 iterations; this resulted in low autocorrelation and a sufficient number of iterations for the model to mix and converge. Convergence of models was assessed by examining trace‐plots to visualize sampling mixing and by assessing effective sample sizes. Further, we plotted predicted random effect values to visually check for normality. We considered estimates of fixed effects to be different from zero when the 95% credible intervals of the posterior distribution did not overlap with zero. These models were fitted with a threshold distribution and a probit link. Residual variance was fixed at one due to the use of binary data.

The first model we fitted used the full data set including all individuals for which we had disease, genetic, and spatial data (*N* = 342), and decomposed variance not accounted for by the fixed effects into two components: additive genetic effects and shared‐environment effects. Additive genetic effects were estimated by fitting the relatedness matrix as a covariance matrix. This was therefore an ‘animal model’, which extends linear mixed effects models to incorporate relatedness information, and partitions phenotypic variance into additive genetic effects and other sources of variance (Kruuk, 2004; Morrissey et al., 2014; Wilson et al., 2010). Shared‐environment effects were estimated by including the home range overlap matrix as an additional covariance matrix, allowing us to estimate the variance associated with individuals sharing the same environment. Prior to fitting the animal model, we ensured that the relatedness matrix and the home range overlap matrix were not correlated with each other and would not affect variance partitioning. The Pearson's correlation between the two matrices was 0.3, which we deemed low enough to not affect parameter estimates derived from the model. Nevertheless, we also fitted the model without the spatial overlap matrix and compared the deviance information criterion (DIC) of the two models to further examine evidence for a shared environment effect. Doing so also enabled us to ensure that fitting the spatial overlap matrix did not influence our heritability estimates. Fitting animal models requires positive definite matrices, and as our relatedness matrix was not positive definite, we calculated the nearest positive definite matrix to our observed relatedness matrix using the corpcor package in R (Schaefer et al., 2013). We subsequently ensured that the original information contained in the relatedness matrix was unchanged by calculating and visualizing the correlation between the observed and the new matrix (*r* ^2^ = .98). The observed spatial overlap matrix was positive definite and therefore did not require transformation.

The second model we ran was used to investigate the extent to which the probability that a koala tests positive for *C. pecorum* may be caused by vertical transmission from mother to offspring (maternal effects). This is necessary because heritability estimates may be inflated when maternal effects are not accounted for (Kruuk & Hadfield, 2007). To do this, we used a subset of the data used for the first model that included only the individuals for which we knew the mother (*N*_ind_ = 195 of *N* = 106 mothers). Although analytically possible, we considered this reduced sample size too small and lacking in statistical power to fully partition variance in disease status into additive genetic effects, maternal effects, and shared environment effects. Instead, we aimed to (1) estimate the extent of maternal effect variance, and (2) ensure that our heritability estimates were not inflated by potential mother‐offspring transmission of disease. To do this, we again fitted the model with the relatedness matrix (as explained above), but here included maternal ID as an additional random effect (instead of the shared environment effect). The maternal ID term estimates the phenotypic variance that is attributed to individuals sharing the same mother, over and above that due to shared additive genetic effects (Thomson et al., 2018; Wilson et al., 2010).

#### 2.7.1 Heritability estimation

We estimated narrow‐sense heritability (*h* ^2^) as the amount of additive genetic variance divided by the total phenotypic variance (i.e., the sum of the different variance components). Because the model was fitted with binary data, all variance estimates of the model were calculated on a latent scale. It is possible to measure heritability on either the latent trait scale or the observed data‐scale, the selection of which depends on inferences being made (Morrissey et al., 2014). Heritability on the latent scale was estimated as $h^{2}=V_{A}/\left(V_{A}+V_{\mathrm{SE}}+V_{e}\right)$, where $V_{A}$ is the additive genetic variance, $V_{\mathrm{SE}}$ is the variance associated with shared environment, and $V_{e}$ is the residual variance, which was fixed at 1. In this case, we also estimated heritability on the observed data‐scale, as this provides parameter estimates which are directly interpretable in relation to the ecology of the species whilst incorporating other factors (i.e., fixed effects and residual variance) which may influence the probability of contracting chlamydia. To convert parameter estimates, we used the qgglmm package in R (de Villemereuil et al., 2016). This package uses estimates of additive genetic variance, phenotypic variance (sum of all random effects variance + residual variance) and the intercept and converts them to the data‐scale, thereby allowing for the calculation of heritability on this scale. We repeated this process for all random effect variances, thus measuring the proportion of phenotypic variance attributed to either shared environment effects or maternal effects (hereafter referred to as intraclass coefficient [ICC]).

## 3 RESULTS

A total of 342 individuals for which we had genetic, spatial and disease status data were included in our final data set. This consisted of 155 males (of which one was a joey <1 year old caught with its mother) and 187 females (of which three were joeys <1 year old caught with their mothers). We were able to detect maternities for 195 individuals, who were the offspring of 106 unique mothers.

**FIGURE 1:**
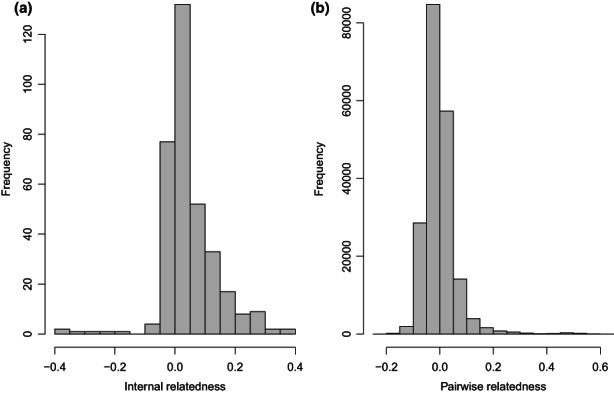
Distribution of (a) internal relatedness of koalas used in analyses, and (b) pairwise relatedness from the matrix used to estimate additive genetic variance. Pairwise relatedness was estimated using Queller and Goodnight (1989) estimate, where, for instance, full‐sibs and parent‐offspring pairs would have a value of 0.5, and half‐sibs would have a value of 0.25.

Out of the total 342 individuals, there were 60 that tested positive at first capture. Observed heterozygosity was estimated as 0.223, within population genetic diversity (also known as expected heterozygosity) was estimated as 0.278, and the population average inbreeding coefficient, *F*_IS_, was estimated as 0.196. The identity disequilibrium proxy (*g*_2_) in our 5007 SNP data set differed significantly from zero (*g*_2_ = 0.013 ± 0.002 bootstrap confidence interval = 0.0101–0.0167, P[*g*_2_ = 0] = 0.001), where permutations = 1000 and bootstraps = 1000. This indicated that our data set met the requirements of variance between individuals in levels of inbreeding, which is required for detecting if there is inbreeding depression in a population. Accordingly, IR varied substantially between individuals, ranging from a minimum estimate of −0.39 (expected when the individuals' parents are outbred) to 0.38 (expected when the individuals' parents are related to one another) (mean IR = 0.05, standard deviation = 0.09, Figure 1a). The variance in pairwise relatedness values was 0.004, with approximately 232 pairs of first‐degree relatives (i.e., parent‐offspring pairs or full siblings) and 680 pairs of second‐degree relatives (see Figure 1b).

**FIGURE 2:**
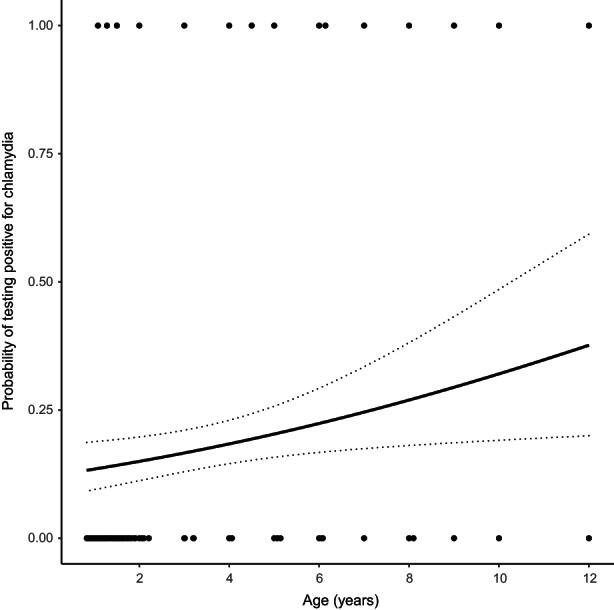
The effect of age on the probability of testing positive for chlamydia. Data points represent the raw data of whether the koala tested positive at first capture. Regression line derived from model estimates represents the mode of the predicted relationship between age and probability of testing positive, and dotted lines are the standard errors for that prediction.

**FIGURE 3:**
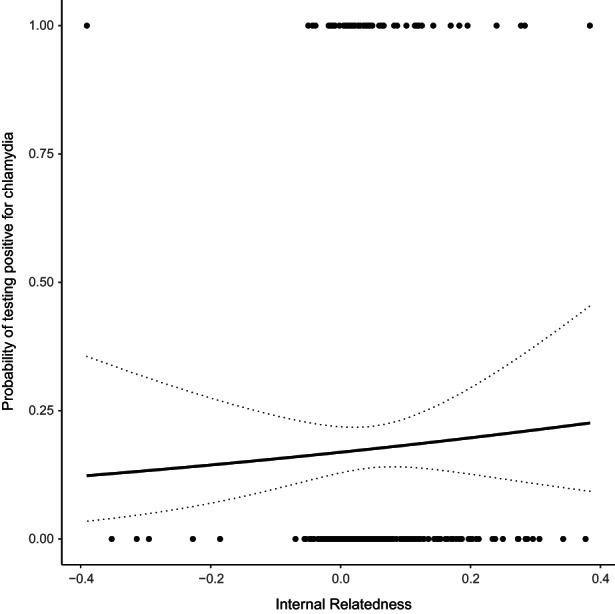
The effect of internal relatedness (IR) of probability of testing positive for chlamydia. Data points represent the raw data of whether the koala tested positive at first capture. Regression line derived from model estimates represents the mode of the predicted relationship between IR and probability of testing positive, and dotted lines are the standard errors for that prediction.

There was no evidence for sex differences in the probability that a koala tested positive for *C. pecorum* (posterior mode *β*_FEMALE_ = 0.037, Table 1). Koalas had a higher probability of testing positive for *C. pecorum* in the breeding season compared to the nonbreeding season (posterior mode *β*_BREEDING_ = 0.623, Table 1). The probability of testing positive for *C. pecorum* also increased with age (posterior mode *β* = 0.145, Table 1, Figure 2). There was no evidence that internal relatedness affected the probability that koalas tested positive for *C. pecorum* (posterior mode *β* = 1.071, Table 1, Figure 3). Furthermore, there was no evidence that the effect of internal relatedness changed with the age of the koala (IR*age interaction, posterior mode *β* = −0.038, Table 1). Together, the lack of an association between IR and disease suggests that there was no evidence for inbreeding depression in this population.

**TABLE 1:** Estimates for both fixed and random effects from a model used to investigate the effect of inbreeding, additive genetic variance (*V*_A_) and variance in shared environment effects (*V*_S_) on the probability of koalas testing positive for *Chlamydia pecorum*

	Posterior distribution			
	Mode	Mean	CI 5%	CI 95%
Fixed effects				
Intercept	−2.332	−2.436	−3.445	−1.383
Sex_FEMALE_	0.037	0.098	−0.402	0.587
Age	0.145	0.147	0.033	0.248
IR	1.071	1.496	−3.402	6.122
Season_BREEDING_	0.623	0.691	0.217	1.122
Age*IR	−0.038	−0.133	−0.179	0.852
Random effects				
*V*_A_				
Latent	1.348	1.575	0.225	2.935
Data‐scale	0.008	0.014	0.003	0.326
*h* ^2^				
Latent	0.573	0.576	0.33	0.737
Data‐scale	0.112	0.142	0.059	0.229
*V*_S_				
Latent	<0.001	0.019	<0.001	0.071
Data‐scale	<0.001	<0.001	<0.001	<0.001
ICC_S_				
Latent	<0.001	0.009	<0.001	0.034
Data‐scale	<0.001	0.002	<0.001	0.008

*Note*: Values in brackets are 95% credible intervals. “Sex” indicates difference in females relative to males; “Age” is the effect in years; “IR” is an individual's internal relatedness value; “Season” is the effect of breeding season relative to nonbreeding; and Age*IR is the interaction. Variance component estimates (for additive genetic variance *V*_A_ and shared environment variance *V*_S_), heritability (*h* ^2^) and the proportion of variance attributed to a shared environment effect (ICC_S_) are all presented on both latent and observed data‐scale. Parameters estimates were converted to the observed data‐scale using the QG_glmm_ package (see Section 2 for details of models). *N* = 324 individuals.

The probability of testing positive for *C. pecorum* was associated with moderate levels of additive genetic variance (*V*_A_) on both latent and observed data scales, whereby the posterior distribution was clearly different from zero on both scales (Table 1, see Figure S1 for posteriors). More specifically, the *V*_A_ for the probability of testing positive for *C. pecorum* was estimated at 1.35 on the latent scale (posterior mode, 95% CI: 0.23–2.93, Table 1), and 0.008 on the observed scale (posterior mode, 95% CI: 0.003–0.33, Table 2). The heritability (*h* ^2^) for the probability of being diseased was estimated at 0.57 on the latent scale (posterior mode, 95% CI: 0.33–0.74), and 0.11 on the observed scale (posterior mode, 95% CI: 0.06–0.23, Table 1).

**TABLE 2:** Estimates for both fixed and random effects from a model used to investigate maternal effects on the probability of koalas testing positive for *Chlamydia pecorum*

	Posterior distribution			
	Mode	Mean	CI 5%	CI 95%
Fixed effects				
β				
Intercept	−2.106	−2.482	−3.58	−1.491
Sex_FEMALE_	−0.044	0.073	−0.438	0.599
Age	0.103	0.12	0.001	0.23
IR	0.961	1.381	−2.935	6.201
Season_BREEDING_	0.767	0.698	0.198	1.222
Age*IR	0.05	−0.134	−1.129	0.927
Random effects				
*V*_A_				
Latent	1.496	1.529	0.316	2.861
Data‐scale	0.009	0.009	0.003	0.013
*h* ^2^				
Latent	0.611	0.532	0.296	0.713
Data‐scale	0.128	0.111	0.071	0.136
*V*_ME_				
Latent	0.005	0.204	<0.001	0.683
Data‐scale	<0.001	0.001	<0.001	0.004
ICC_ME_				
Latent	0.001	0.073	0.001	0.237
Data‐scale	<0.001	0.015	<0.001	0.049

*Note*: Values in brackets are 95% credible intervals. “Sex” indicates difference in females relative to males; “Age” is the effect in years; “IR” is an individual's internal relatedness value; “Season” is the effect of breeding season relative to nonbreeding; and Age*IR is the interaction. Random effect variance estimates, genetic heritability (*h* ^2^) and proportion of variance attributed to maternal effects (ICC_ME_) are all presented on both latent and observed data‐scale. Parameters estimates were converted to the observed data‐scale using QG_glmm_ package. *N* = 195 individuals, for whom maternity was known, born to 106 mothers.

We found no evidence that the probability of testing positive for *C. pecorum* was associated with variance in shared environment effects (*V*_S_). The posterior distribution for *V*_S_ (measured using the home range overlap matrix) was very low and bordered zero on both the latent and observed data scales (latent scale; posterior mode = <0.001, 95% CI: <0.001–0.071: observed data scale; posterior mode = <0.001, 95% CI: <0.001–<0.001, Table 1). This represented <1% of variance in probability of being diseased on both latent and data scales. Furthermore, the inclusion of *V*_S_ in the model did not improve the fit of the model (DIC *V*_A_ + *V*_S_ = 278.69, DIC *V*_A_ = 277.88). Finally, *V*_A_ estimates did not vary qualitatively between models with and without the home range overlap matrix, suggesting that its inclusion did not affect our reported estimates of *h* ^2^ (see Table S1).

Using a subset of the data that included only individuals for which we knew the identity of their mothers (*N* = 195), we found only a small maternal effect variance (*V*_ME_) in the probability of testing positive for *C. pecorum*. More specifically, the posterior mode of *V*_ME_ was estimated as 0.005 on the latent scale, and <0.001 on the observed data‐scale, and the lower tail of the posterior distribution bordered zero on both scales (see Table 2 and Figure S2 for full posteriors). This corresponded to a low proportion of phenotypic variance attributable to maternal effects (ICC_ME_), with no indication that ICC_ME_ was statistically different from zero (Table 2). Given the low sample size, it may be that we lacked the statistical power to separate *V*_A_ from *V*_ME_ within a single model. However, when running a model without estimating *V*_A_ (i.e., without the relatedness matrix), we found that *V*_ME_ did not differ (qualittively) to that estimated when fitting both simultaneously (see Tables S1 and S2). Moreover, the DIC was lowest for the model containing just the relatedness matrix, and highest for the model containing just the maternal effects (DIC: *V*_A_ + *V*_ME_ = 277.66, *V*_ME_ = 314.20, *V*_A_ = 140.64). Together, this suggests that maternal effects explained little to none of the phenotypic variance in the probability of individuals testing positive for *C. pecorum*. Finally, estimates for *V*_A_ and for *h* ^2^ did not differ qualitatively between the model containing the maternal effect versus without, suggesting that our reported heritability estimates (Table 1) were not inflated by maternal effects that were unaccounted for.

## 4 DISCUSSION

Our results showed that aging and breeding season increased the probability of testing positive for *C. pecorum* in a wild koala population, but there was no evidence of effects of either sex or inbreeding on disease. We did, however, find evidence for heritable genetic variation in susceptibility to disease (*h* ^2^ = 0.11). We discuss these results in more detail below.

### 4.1 Absence of inbreeding depression

Inbreeding depression can be defined as heterozygosity‐fitness correlations (Grueber et al., 2008) and, using this approach, we found no evidence of inbreeding depression in disease (a key fitness trait) in this koala population. Inbreeding depression is difficult to investigate in wild populations, not least because obtaining necessary data sets (including pedigrees and/or genome‐wide markers and fitness measures) is both costly and labour intensive. However, given the potential consequences of inbreeding depression on population growth and survival (Hedrick & Kalinowski, 2000), identifying the risk it poses to populations is critical to implementing informed management. Interestingly, empirical evidence of an association between disease and inbreeding in wild populations is mixed, with effects varying both between and within species (Benton et al., 2018; Coltman et al., 1999; Reid et al., 2003; Townsend et al., 2009), and with pathogen type (Acevedo‐Whitehouse et al., 2003). In koalas, evidence to date for inbreeding depression is also mixed. For instance, inbreeding depression was found when comparing cryptorchidism levels in island to mainland populations (Seymour et al., 2001), but the relationship was not evident at the individual level (Cristescu et al., 2009), and did not correspond to decreased fitness, as the most inbred populations were also experiencing exponential population growth (Masters et al., 2004).

The effects of inbreeding on individual or population fitness can be dependent on environment, age, sex and the genetic constitution of populations (Hedrick & Kalinowski, 2000; Marjamäki et al., 2021). Interestingly, it has been suggested that koalas may have a reduced susceptibility to inbreeding depression, due to low historical effective population sizes and the elimination of recessive deleterious alleles (Wilmer et al., 1993). Although we did not detect an age‐dependent effect of inbreeding in our analyses, there may have been other context dependent effects of inbreeding that we did not measure. As such, although the lack of inbreeding depression could reduce concerns for this population, our results provide no guarantee of future resilience to chlamydial disease, especially as the context in which koalas live rapidly changes under continued environmental degradation.

### 4.2 Heritability of susceptibility to disease in the wild

Measures of heritability for disease susceptibility in wild populations are relatively rare: for example, only between 0.03% and 2.5% of studies of heritability estimates of traits in wild populations were related to disease (Postma, 2014; Wood et al., 2016). However, some recent studies have found that there may be considerable additive genetic variation associated with disease susceptibility in the wild. For instance, heritability of infection risk was found to be 12% for *Mycobacterium bovis* in adult European badgers (Marjamäki et al., 2021) and 55% for *Mycoplasma ovipneumoniae* in bighorn sheep (Martin et al., 2021). Interestingly, we found relatively low environmental effect variance for disease in this population, explaining <1% of the total variance. The low shared‐environment effect variance found here may reflect the small size of the study area that this population inhabits (of only 13 km length, and several 100 m width), and therefore relative homogeneity in environmental conditions for all koalas. Nevertheless, considerable variation in susceptibility to disease remains unexplained (in the residual variance), suggesting that there are other environmental factors influencing disease in this population that were not captured by the shared environment effect. Moreover, additive genetic effects in disease are probably not homogeneous across ages (Marjamäki et al., 2021). We were not able to formally investigate this, owing to low statistical power at different ages, but this would be an interesting and valuable follow‐up study.

Narrow sense heritability (*h* ^2^), as calculated in this study, can be used to predict the response to selection across generations (Walsh & Lynch, 2018), and can therefore be used to predict outcomes for populations facing disease outbreaks (Golas et al., 2021). Heritability is a population‐level parameter which depends on population‐specific factors (e.g., allele frequencies or the effects of gene variants), traits, and variation due to environmental factors (Harrisson et al., 2014). Together with recent findings suggesting that polymorphisms in immune genes play an important role in koalas' ability to resolve a chlamydial infection (Silver et al., 2022), the additive genetic variance for susceptibility to infection suggests there may be some potential for koalas to respond adaptively to the presence of the pathogen. Given the degree of heritability found in this population, the immediate question is: why have koalas in this population not yet adapted to be resilient to chlamydia?

There are several possible reasons for the maintenance of genetic variance in disease susceptibility in this population. First, genetic variance may be maintained through antagonistic pleiotropy with traits associated with other fitness components (Cotter et al., 2004). Second, predation (predominantly by dogs) accounted for about 63% of all mortality in this population (Beyer et al., 2018) and could therefore have imposed a stronger selective pressure than disease. Third, koalas are hypothesized to have both their own chlamydia species and others more recently transmitted from livestock over the last 200 years (Timms, 2005), so – whilst there is not yet any empirical support for this hypothesis – a possible explanation for the current virulence of chlamydia is that new strains have recently been introduced, and there has not been sufficient time for adaptation of resistance to these new strains. Finally, pathogens also adapt in response to host resistance and/or tolerance, and theoretical models suggest that maintenance in genetic variation in resistance to pathogens may be explained by a continuous process of host‐pathogen coevolution (Best et al., 2008; Boots et al., 2009; Mazé‐Guilmo et al., 2014).

### 4.3 Can our findings be used to improve koala population management?

Management of the threat of chlamydia to koalas has been difficult thus far: vaccine development is promising, but still a work in progress (Khan et al., 2016), and treatment is effective only in certain koalas or for a limited time (Robbins et al., 2018). Therefore, although treatment of isolated koala populations can decrease chlamydia prevalence and reverse population decline (Beyer et al., 2018), in nonisolated populations into which diseafGruebersed animals may immigrate, maintaining effective treatment will probably require intensive and repeated interventions. Heritability of susceptibility to disease found here might open the door to enhancing adaptation through genetic rescue or artificial selection (van Oppen et al., 2015). Individuals that are the most resilient to disease could be identified and selected for breeding programmes and/or translocations into struggling populations (Gienapp et al., 2017; Gonzalez et al., 2013). Strategies such as this are not without risk or concerns. In general, these centre around the potential decrease in genetic diversity that can be associated with selection, ethical and moral concerns regarding anthropogenic interference with natural processes, or whether resources might be better allocated to more urgent or productive causes (Kardos & Shafer, 2018; Kosch et al., 2022). One also needs to ensure that efficient selection in the face of current challenges does not compromise resilience to uncertain future challenges (Harrisson et al., 2014).

Disease management is one of the greatest challenges of wildlife management. It is hard to gain knowledge of diseases in the wild, although noninvasive methods are promising. For example, surveillance can be enhanced by the use of detection dogs and pathogen detection from scats (for examples with koalas and chlamydia see (Cristescu et al., 2015, 2019, 2020)). Ultimately, disease management options must be supported by decision makers and the public, and usually need to balance a multitude of factors – including risk and ethical perception, cost, concern and tolerance about (perceived or real) impacts on wildlife, livestock and human health, values and social acceptability of management actions as well as scientifically expected outcomes and uncertainties. Incorporating information about genetic susceptibility for disease when assessing risk to populations and in conservation planning is therefore important in ensuring we implement effective management strategies.

## AUTHOR CONTRIBUTIONS

Romane H. Cristescu, Kasha Strickland and Céline H. Frère conceived and designed the research; Deidre de Villiers collected data; Anthony J. Schultz and Kasha Strickland analysed SNP data to generate relatedness and internal relatedness information; Kasha Strickland analysed the data, with input from Loeske E. B. Kruuk and Céline H. Frère; Kasha Strickland and Romane H. Cristescu wrote the manuscript, and all authors contributed to reviewing and editing.

## CONFLICT OF INTEREST

All authors declare no conflict of interests.

## Supporting information


Appendix S1

## Data Availability

All data have been made available in the Dryad repository at (https://doi.org/10.5061/dryad.np5hqbzwx; Strickland et al., 2022).
