# Supplementary material for: Susceptibility to a sexually transmitted disease in a wild koala population shows heritable genetic variance but no inbreeding depression
Source: Mol Ecol. 2022 Sep 12;31(21):5455–67. doi: 10.1111/mec.16676 (PMC9826501; doi:10.1111/mec.16676)
Supplement: Supplementary file 1 — Appendix S1 [file MEC-31-5455-s001.pdf]

MOLECULAR ECOLOGY

Supplemental Information for:

Susceptibility to a sexually transmitted disease in a wild koala population shows heritable genetic variance but no inbreeding depression

Romane H Cristescu§, Kasha Strickland§*, Anthony J Schultz, Loeske E B Kruuk, Deidre de Villiers, Céline H Frère

§ These authors contributed equally to the work

* Corresponding author: kasha.strickland@ed.ac.uk

Table of Contents:

**Figure S1**	Page 2 - 4
**Figure S2**	Page 5 - 7
**Table S1**	Page 8
**Table S2**	Page 9

MOLECULAR ECOLOGY

Figure S1. Trace and density plots showing posterior distribution of parameter estimates for a model used to investigate the effect of internal relatedness, additive genetic effects, and shared environment effect on probability of testing positive for *C. pecorum*. "IR_new" is internal relatedness, "Name" refers to the additive genetic effects, "Name2" refers to the shared environment effect. Residual variance ("Units") was fixed at one.

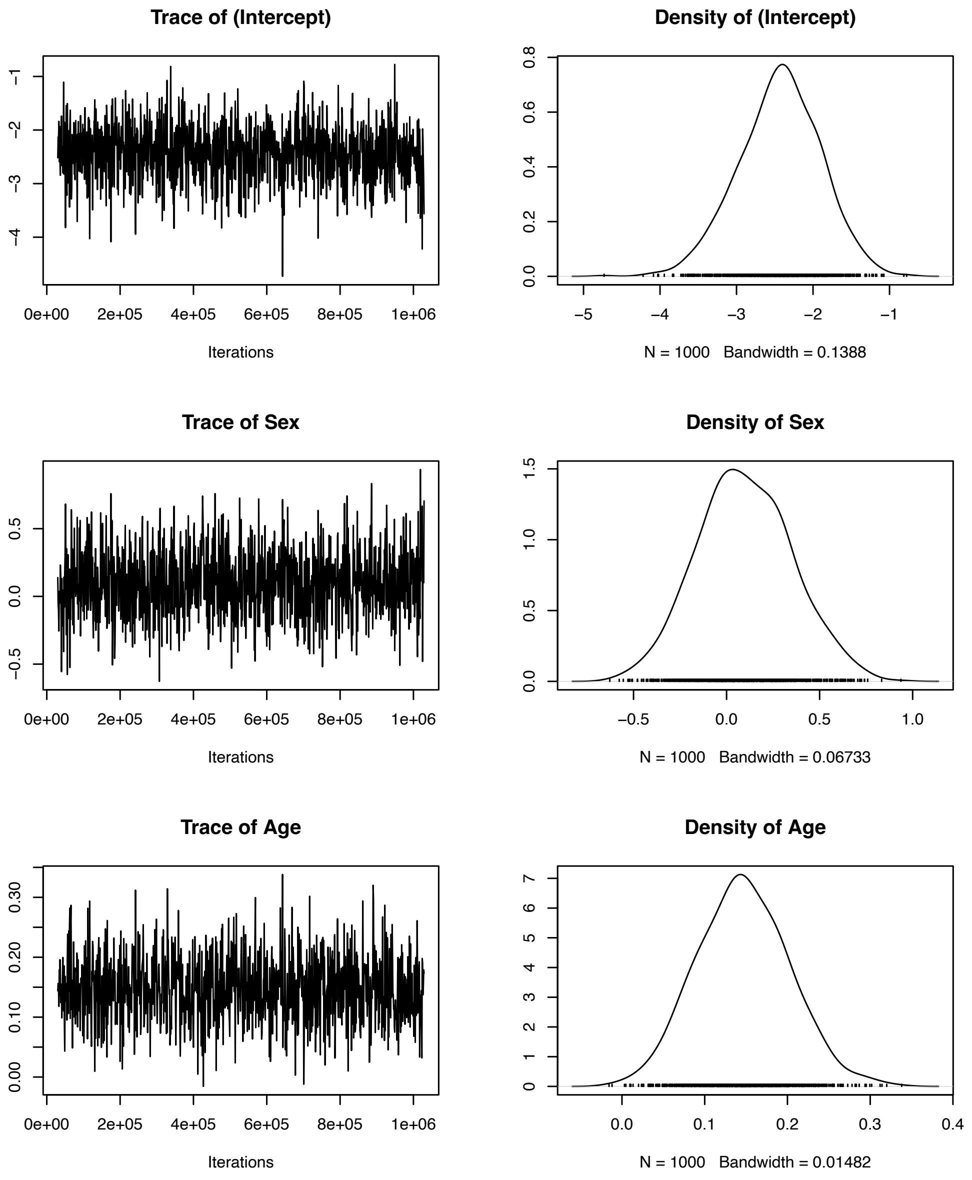

MOLECULAR ECOLOGY

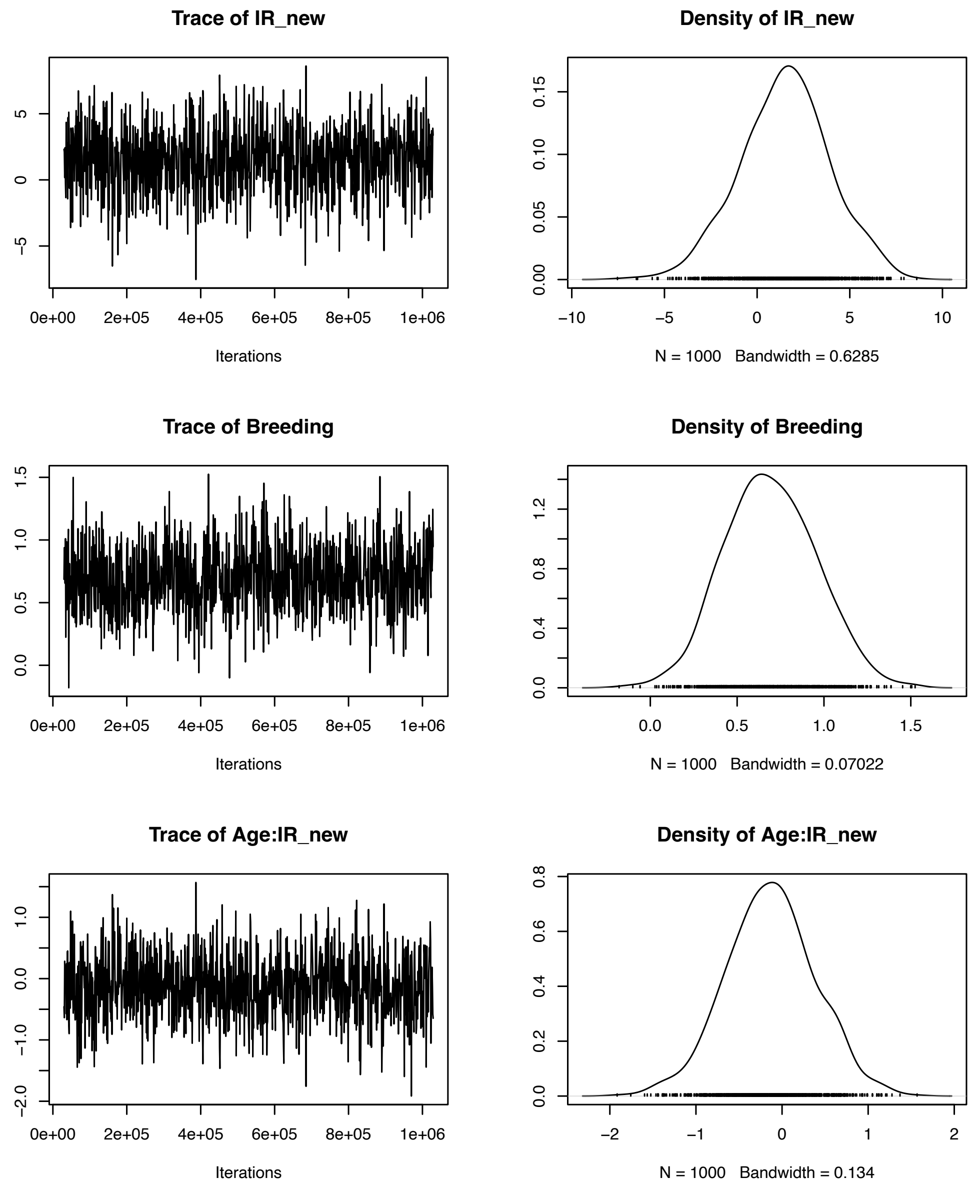

MOLECULAR ECOLOGY

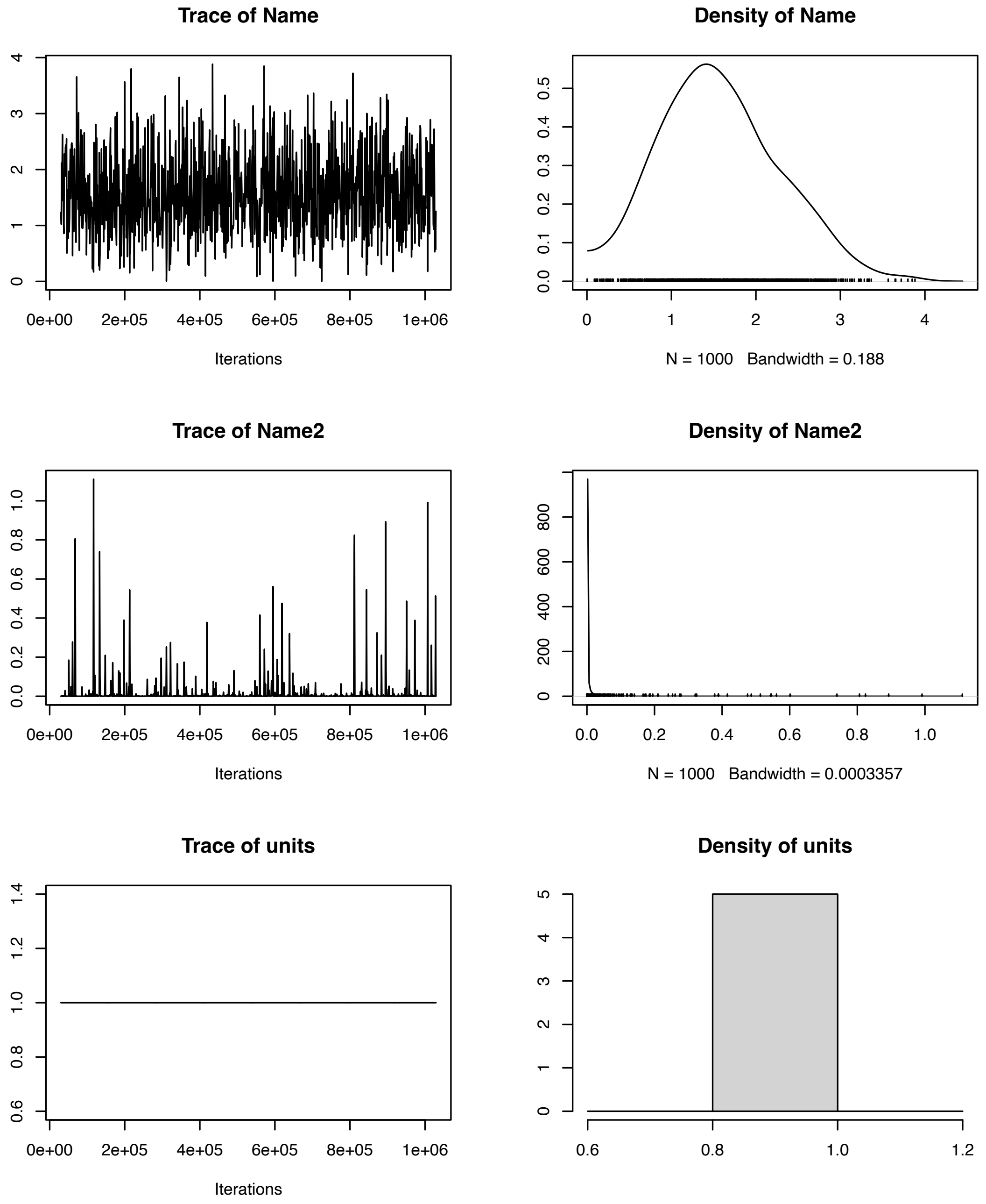

MOLECULAR ECOLOGY

Figure S2. Trace and density plots showing posterior distribution of parameter estimates for a model used to investigate the effect of internal relatedness, additive genetic effects, and maternal effect on probability of testing positive for *C. pecorum*. “IR_new” is internal relatedness, “Name” refers to the additive genetic effects, “maternal_id” refers to the shared environment effect. Residual variance (“Units”) was fixed at one.

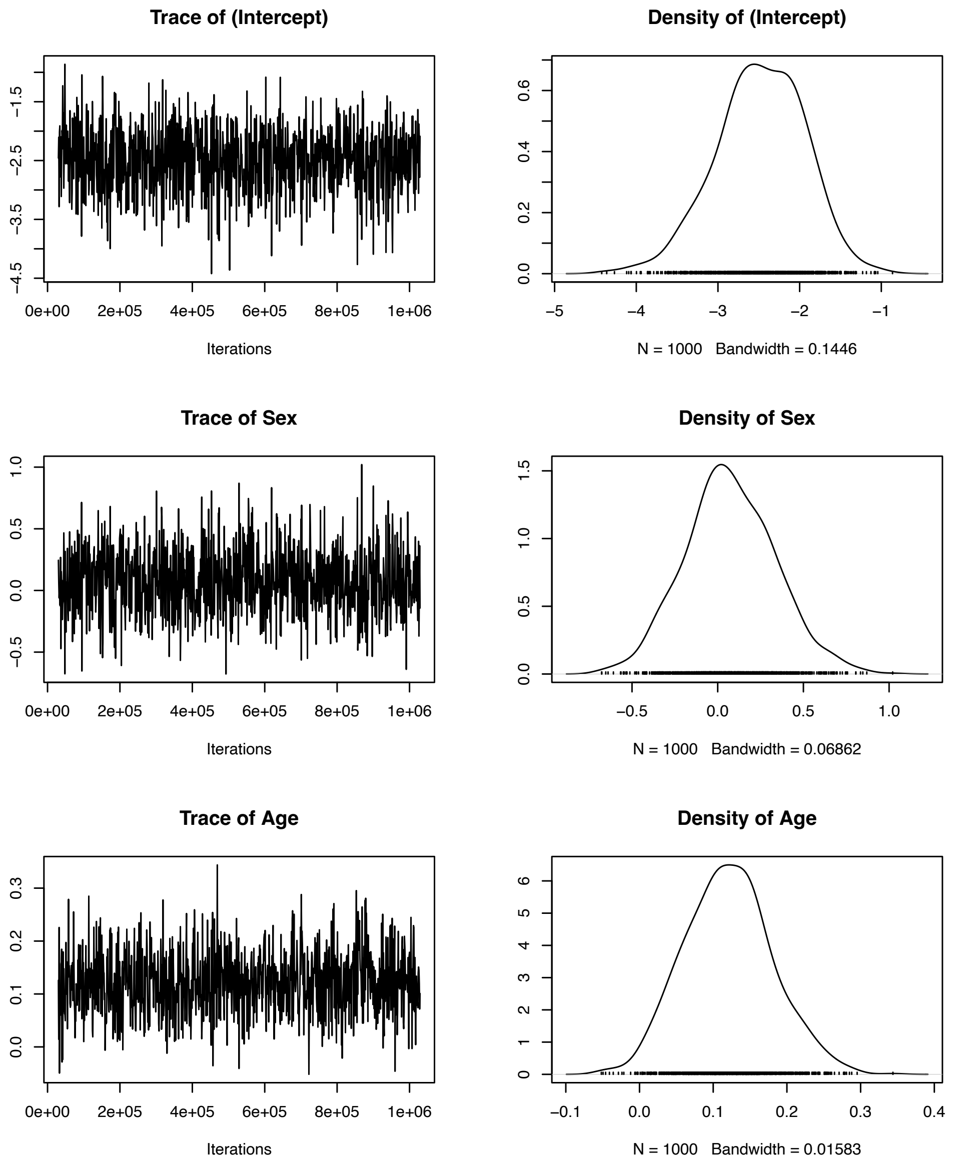

MOLECULAR ECOLOGY

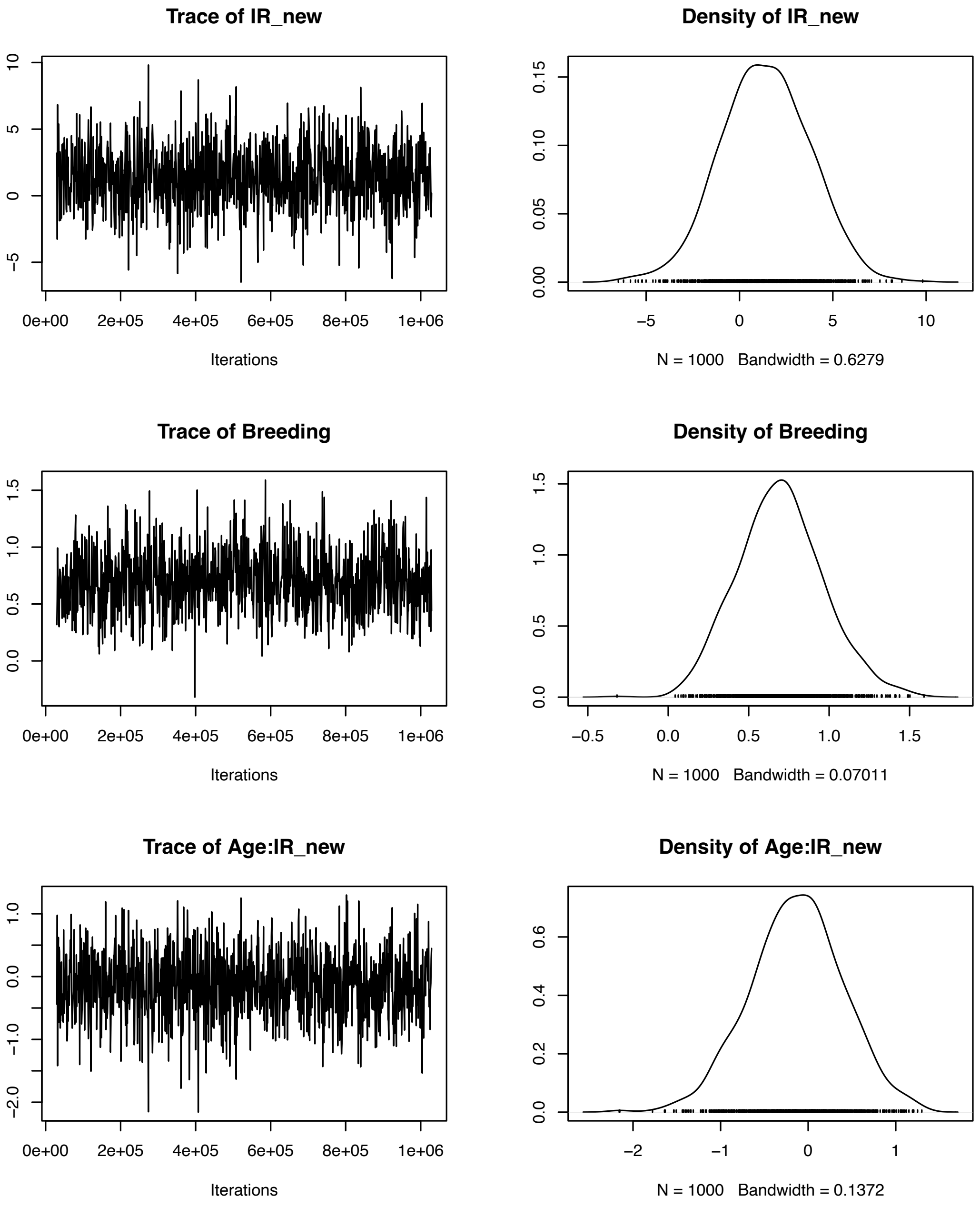

MOLECULAR ECOLOGY

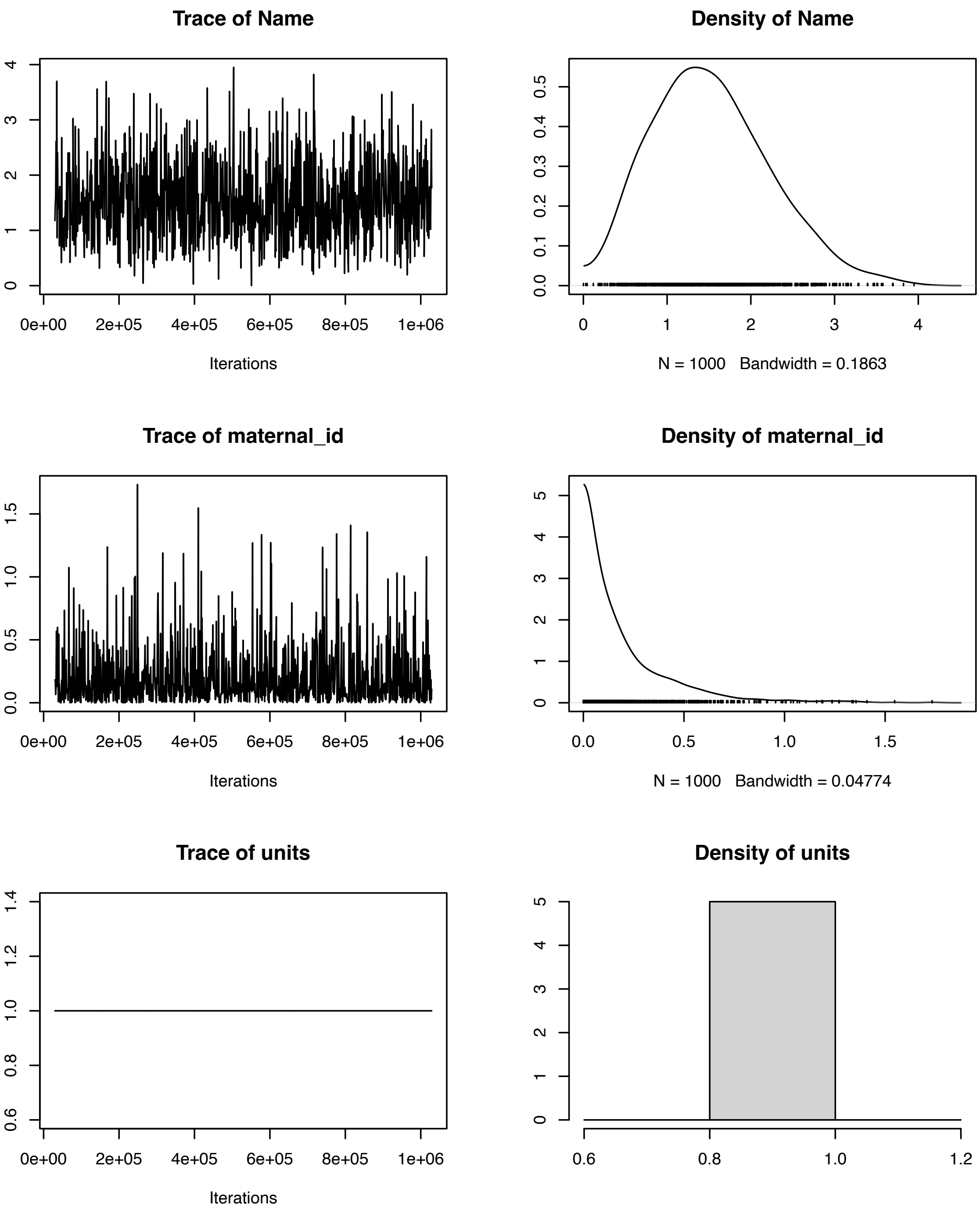

Table S1. Estimates for both fixed and random effects from a model used to investigate additive genetic effects for the probability of koalas testing positive for *Chlamydia pecorum*. Values in brackets are 95% credible intervals. Random effect variance estimates and genetic heritability (h^2) are both presented on both latent and observed data-scale are presented. Parameters estimates were converted to the observed data-scale using QG_{GLMM} package.

			Posterior distribution			
			mode	*mean*	*CI5%*	*CI95%*
Fixed effects	β	Intercept	-2.292	-2.439	-3.441	-1.4
		Sex_{FEMALE}	0.094	0.084	-0.396	0.59
		Age	0.145	0.152	0.045	0.262
		IR	1.527	1.427	-3.293	5.912
		$Season_{BREEDING}$	0.601	0.678	0.119	1.2
		Age*IR	-0.057	-0.136	-1.189	0.829
Random effects	Latent	V_A	1.614	1.627	0.339	2.957
	Data-scale	V_A	0.006	0.015	0.003	0.034
	Latent	h^2	0.634	0.588	0.336	0.741
	Data-scale	h^2	0.101	0.145	0.066	0.243

MOLECULAR ECOLOGY

Table S2. Estimates for both fixed and random effects from a model used to investigate maternal effects for the probability of koalas testing positive for *Chlamydia pecorum*. Values in brackets are 95% credible intervals. Random effect variance estimates presented on both latent and observed data-scale are presented. Parameters estimates were converted to the observed data-scale using QG_{GLMM} package.

			Posterior distribution			
			mode	*mean*	*CI5%*	*CI95%*
Fixed effects	β	Intercept	-1.779	-1.861	-2.592	-1.194
		Sex_{FEMALE}	0.081	0.121	-0.204	0.477
		Age	0.084	0.069	-0.006	0.141
		IR	0.671	1.253	-1.645	4.754
		$Season_{BREEDING}$	0.59	0.516	0.179	0.869
		Age*IR	-0.061	-0.162	-0.835	0.431
Random effects	Latent	V_{ME}	0.004	0.221	<0.001	0.804
	Data-scale	V_{ME}	<0.001	0.003	<0.001	0.011
	Latent	ICC_{ME}	0.003	0.151	0.003	0.445
	Data-scale	ICC_{ME}	0.001	0.031	0.001	0.097
